# Supplementary material for: Predictors of Suicide Risk and Mental Health Outcomes among Hong Kong Veterinarians: A Cross-Sectional Study
Source: Behav Sci (Basel). 2023 Sep 15;13(9):770. doi: 10.3390/bs13090770 (PMC10526002; doi:10.3390/bs13090770)
Supplement: Supplementary file 1 [file behavsci-13-00770-s001.zip › behavsci-2552068-supplementary.pdf]

Manuscript title

Professional quality of life and mental health among Hong Kong veterinarians: a quantitative pilot study

Authors names, affiliation, and address

CHAN, Camille K.Y.[1]

ORCID: 0000-0003-2971-7542

WONG, Paul W.C.[1]

paulw@hku.hk

ORCID: 0000-0003-3388-6285

[1] Department of Social Work and Social Administration, The University of Hong Kong

Corresponding author

CHAN, Camille K.Y. (cchanky@hku.hk)

5/F, Department of Social Work and Social Administration, Jockey Club Tower, The Centennial Campus, The University of Hong Kong, Pokfulam, Hong Kong

(+852) 6683 8563

Supplementary Table S1. Data completion rate

	**Participants** (N=56)
**Mental health status**	
PHQ-9 total score	51 (91.1)
GAD-7 total score	51 (91.1)
SBQ-R total score	50 (89.3)
**ProQOL**	
CS score	56 (100.0)
BO score	56 (100.0)
STS score	56 (100.0)

Note:
PHQ-9 = Patient Health Questionnaire–9
GAD-7 = Generalised Anxiety Disorder–7
SBQ-R = Suicidal Behaviors Questionnaire-Revised
ProQOL = Professional Quality of Lift scale
CS = compassion satisfaction
BO = burnout
STS = secondary traumatic stress

Supplementary Table S2. SBQ-R results.

		Mean (SD)	Median	IQR	At risk of suicide (SBQ-R >7)
**Total respondents**	N=50	5.8 (3.4)	5.0	3.0-7.0	11 (22.0)
**Lifetime suicide-risk ideation**					
Non-Risk / Non-Suicidal	n=25	3.6 (1.0)	3.0	3.0-5.0	0 (0.0)
Suicide-risk ideation					
Brief passing thought	n=17	6.2 (2.0)	6.0	5.0-7.0	3 (6.0)
Suicide plan without intent	n=5	11.2 (2.4)	11.0	10.0-13.0	5 (10.0)
Suicide plan with intent	n=2	14.5 (3.5)	14.5	12.0-17.0	2 (4.0)
Suicide attempt without intent	n=1	8 (NA)	NA	NA	1 (2.0)
**Past 12 months suicide ideation**					
Never	n=33	3.9 (1.2)	3.0	3.0-5.0	0 (0.0)
Once	n=8	7.6 (2.1)	7.5	6.0-9.0	4 (8.0)
Twice	n=4	7.8 (1.7)	7.5	6.5-9.0	2 (4.0)
3-4 times	n=2	12.0 (1.4)	12.0	11.0-13.0	2 (4.0)
5 times or more	n=3	14.3 (2.5)	14.0	13.0-15.5	3 (6.0)
**DISCLOSURE OF POSSIBLE SUICIDE ATTEMPT**					
No	n=41	4.6 (2.0)	4.0	3.0-6.0	4 (8.0)
Once, without suicide intent	n=4	8.3 (2.1)	8.5	6.5-10.0	2 (4.0)
More than once, without suicide intent	n=4	12.3 (1.5)	12.0	11.0-13.5	4 (8.0)
More than once, with suicide intent	n=1	21 (NA)	NA	NA	1 (2.0)
**LIKELIHOOD OF SUICIDE IN THE FUTURE**					
Never / No chance	n=33	4.0 (1.3)	3.0	3.0-5.0	1 (2.0)
Rather unlikely	n=9	7.0 (1.9)	7.0	6.0-8.0	3 (6.0)
Unlikely	n=7	10.9 (2.6)	11.0	10.0-12.5	6 (12.0)
Likely	n=0	NA	NA	NA	NA
Very likely	n=1	21 (NA)	NA	NA	1 (2.0)
**SUICIDE RISKS**					
Not at-risk of suicide	n=39	4.3 (1.4)	4.0	3.0-5.0	39 (78.0)
At-risk of suicide	n=11	11.1 (2.8)	11.0	9.0-12.5	11 (22.0)

[a] Percentage based on valid sample. IQR – Tukey's Hinges interquartile range. SD – standard deviation. N – Total number of respondents. n – Number of respondents in the sub-group. SBQ-R – Suicidal Behaviours Questionnaire-Revised

Supplementary Table S3. Cross-tabulations of ProQOL and mental health.

			BURNOUT			STS			CS		
	**N**	**n**	**Low**	**Moderate**	**High**	**Low**	**Moderate**	**High**	**Low**	**Moderate**	**High**
**All participants**	56	56	13	41	2	19	37	0	2	43	11
**At-risk of suicide**	50	11	1	10	0	1	10	0	0	11	0
**Current suicidal ideation**	51	10	0	9	1	1	9	0	0	10	0
**Depressed**	56	15	0	14	1	0	15	0	0	15	0
**Anxious**	56	15	0	14	1	1	14	0	0	14	1

N – Total number of respondents. n – Number of respondents in the sub-group.

Supplementary Questionnaire S1. Questionnaire

Mental health of veterinarians in Hong Kong

Consent:

I understand the procedures described above and agree to participate in this study.	checkbox
I understand the data collected cannot be withdrawn once provided due to unfeasibility to link anonymity data collected to individual participants.	checkbox

Screener

S1. Are you a veterinary profession who are registered with the Veterinary Surgeon Board of Hong Kong?

Yes	Radio button	Continue
No	Radio button	**Terminate**

S2. Which of the below best describes your registration listed in the Veterinary Surgeon Board of Hong Kong?

Veterinarian	Radio button	Continue
Veterinary Surgeon / Specialists	Radio button	Continue
Others (Please specify)	Textbox	Continue
Refused	Radio button	**Terminate**

S3. Are you currently practicing in Hong Kong?

Yes	Radio button	Continue
No	Radio button	**Terminate**

Main Questionnaire

Section A: We would like to start with understanding your practice background.

A1. What best describes your medical specialty?

Veterinarian (Cats and/or dogs)	Radio button
Veterinary (Small mammals)	Radio button
Veterinary (Reptiles and exotics)	Radio button
Lab veterinarian	Radio button
Equine veterinarian	Radio button
Zoologic veterinarian	Radio button
Farm veterinarian	Radio button
Others (Please specify)	Textbox
Refuse	Radio button

A2. When did you receive your earliest qualification as a veterinarian?

Less than 2 years ago	Radio button
2 to less 5 years ago	Radio button
5 to less than 8 years ago	Radio button
8 to less than 12 years ago	Radio button

12 to less than 16 years ago	Radio button
16 to less than 20 years ago	Radio button
20 to less than 25 years ago	Radio button
More than 25 years	Radio button
Refuse	Radio button

A3a. Have you practiced in locations other than Hong Kong (please exclude your residency)?

Yes	Radio button
No	Radio button

A3b. How many years in total have you practiced in locations other than Hong Kong?

Less than 4 years	Radio button
4 to less than 10 years	Radio button
More than 10 years	Radio button

A4. Do you require translation help when consulting Cantonese speaking clients?

Yes	Radio button
No	Radio button

A5. Which of the following best describe your employment status?

Full time	Radio button
Part time / Locum	Radio button
Others (please specify)	Textbox

A6. Which of the following best describe your work hours?

Mostly between 08:00-20:00	Radio button
Mostly between 20:01-07:59	Radio button
A combination of day-shifts and night-shifts	Radio button
Others (please specify)	Textbox

A7. Which of the following best describe your seniority?

Clinic Owner/ Partner	Radio button
(Non-owner) Managerial level / Key decision makers	Radio button
Employee	Radio button
Intern	Radio button
Others (please specify)	Textbox

A8a. Which of the below best describe your workplace?

Single owner or partnership veterinary clinic / hospital	Radio button
Chained/Group veterinary clinic / hospital	Radio button
Non-government organisation	Radio button
Academic research	Radio button
Government and/or government laboratories	Radio button
Private laboratories	Radio button
Others (please specify)	Textbox

Refuse	98

A8b. Which of the following best describe your clinic role?

General Practice	Radio button
Emergency Medicine	Radio button
Specialist Services	Radio button
Physiotherapy	Radio button
Traditional Chinese Medicine (TCM) or Acupuncture	Radio button
Non-TCM herbal therapy, homeopathy, or reiki	Radio button
Other (Please specify)	Textbox
Refuse	98

A8c. On average, how many hours per week would you be the sole charge of the clinic and in isolation as a vet in your workplace?

I'm never the only vet on duty in my workplace	Radio button
Less than 8 hours per week	Radio button
8 to less than 16 hours per week	Radio button
16 to less than 25 hours per week	Radio button
More than 25 hours per week	Radio button
I'm always the only vet on duty	Radio button

Q8d. How many hours do you work in an average week?

Numeric

A9. Which of the below best describe the owner(s) of your workplace?

Owned by the government corporate	Radio button
Owned by corporate or business investors	Radio button
Owned by veterinarians	Radio button
Owned by both business investors and veterinarians	Radio button
Others (please specify)	Textbox
Refuse	Radio button

A10. In the past 6 months, which district did you spend most of your time practicing?

Hong Kong Island	
Central and Western	Radio button
Eastern	Radio button
Southern	Radio button
Wan Chai	Radio button
Kowloon	
Kowloon City	Radio button
Kwun Tong	Radio button
Sham Shui Po	Radio button
Yau Tsim Mong	Radio button
Wong Tai Sin	Radio button

New Territories	
Kwai Tsing	Radio button
North	Radio button
Outlying Islands	Radio button
Sai Kung	Radio button
Sha Tin	Radio button
Tai Po	Radio button
Tsuen Wan	Radio button
Tuen Mun	Radio button
Yuen Long	Radio button
Refuse	Radio button

A11. What forms of official complaint(s) have you ever received from clients?

Complaints made to the Veterinary Surgeon Board of Hong Kong	Radio button
Complaints made to the Agriculture, Fisheries and Conservation Department (AFCD)	Radio button
Complaints made to the Consumer Council	Radio button
Other forms of legal proceedings	Radio button
None	Radio button
Refuse	Radio button

A12. Are you covered by professional indemnity insurance in your workplace?

Yes	Radio button
No	Radio button
Not sure	Radio button

Section Ba: Demand-Control-Support Questionnaire (DCSQ)

1	2	3	4
Strongly disagree	Disagree	Agree	Strongly agree

	1	2	3	4
Bi1. Do you have to perform your work tasks quickly?				
Bi2. Do you have to work intensely? (In other words, produce a lot in little time)				
Bi3. Does your work require too much from you?				
Bi4. Do you have enough time to perform all the tasks of your job?				
Bi5. Does you work usually involve contradictory or discordant requirements?				
Bii1. Is it possible for you to learn new things through your work?				
Bii2. Does your work require much ability or specialized knowledge?				
Bii3. Does you work require you to show initiative?				
Bii4. In your job, do you have to repeat the same tasks many times?				
Bii5. Can you choose HOW to do your job?				
Bii6. Can you choose WHAT to do in your job?				
Biii1. Is there a calm and pleasant environment where you work?				
Biii2. At work, do you have a good relationship with each other?				

Biii3. Can you rely on the support of your coworkers?					
Biii4. If you are not having a good day, do your coworkers understand you?					
Biii5. At work, do you have a good relationship with your superiors?					
Biii6. Do you like to work with your coworkers?					

Section Bb: Workplace and career

1	2	3	4	5
Strongly Disagree	Disagree	Neither agree nor disagree	Agree	Strongly Agree

To what extend would you agree statements below?

	1	2	3	4	5
B1. Veterinary medicine is a good career choice.					
B2. There is excessive administrative duties and managerial aspects working as a vet.					

Section C: Professional Quality of Life Scale (ProQOL)

When you *help* people and animals, you have direct contact with their lives. As you may have found, your compassion for those you *help* can affect you in positive and negative ways. Below are some questions about your experiences, both positive and negative, as a *veterinarian*. Consider each of the following questions about you and your current work situation. Select the number that honestly reflects how frequently you experienced these things in the *last 30 days*.

1	2	3	4	5
Never	Rarely	Sometimes	Often	Very often

In the last 30 days…

	1	2	3	4	5
C1. I am happy.					
C2. I am preoccupied with more than one client and/or patient I help.					
C3. I get satisfaction from being able to provide care for others.					
C4. I feel connected to others.					
C5. I jump or am startled by unexpected sounds.					
C6. I feel invigorated after working with those I provide care for.					
C7. I find it difficult to separate my personal life from my life as a veterinarian.					
C8. I am not as productive at work because I am losing sleep over traumatic experiences of a client and/or patient that I provide care for.					
C9. I think that I might have been affected by the traumatic stress of those I provide care for.					
C10. I feel trapped by my job as a veterinarian.					
C11. Because of my role as a veterinarian, I have felt "on edge" about various things.					
C12. I like my work as a veterinarian.					
C13. I feel depressed because of the traumatic experiences of the client					

and/or patient I provide care for.					
C14. I feel as though I am experiencing the trauma of client and/or patient I have provide care for.					
C15. I have beliefs that sustain me.					
C16. I am pleased with how I am able to keep up with veterinary techniques and protocols.					
C17. I am the person I always wanted to be.					
C18. My work makes me feel satisfied.					
C19. I feel worn out because of my work as a veterinarian.					
C20. I have happy thoughts and feelings about those I provide care for and how I could help them.					
C21. I feel overwhelmed because my case workload seems endless.					
C22. I believe I can make a difference through my work.					
C23. I avoid certain activities or situations because they remind me of frightening experiences of the client and/or patient I help.					
C24. I am proud of what I can do to provide care for.					
C25. As a result of my care, I have intrusive, frightening thoughts.					
C26. I feel "bogged down" by the system.					
C27. I have thoughts that I am a "success" as a veterinarian.					
C28. I can't recall important parts of my work with trauma victims.					
C29. I am a very caring person.					
C30. I am happy that I chose to do this work.					

NEXT PAGE; ASK ALL
Section D: Self-compassion Scale (SCS)

HOW I TYPICALLY ACT TOWARDS MYSELF IN DIFFICULT TIMES
Please read each statement carefully before answering.
To the left of each item, indicate how often you behave in the stated manner, using the following scale:

1	2	3	4	5
Almost never				Almost always

	1	2	3	4	5
D1. I'm disapproving and judgmental about my own flaws and inadequacies.					
D2. When I'm feeling down, I tend to obsess and fixate on everything that's wrong.					
D3. When things are going badly for me, I see the difficulties as part of life that everyone goes through.					
D4. When I think about my inadequacies, it tends to make me feel more separate and cut off from the rest of the world.					
D5. I try to be loving towards myself when I'm feeling emotional pain.					
D6. When I fail at something important to me, I become consumed by feelings of inadequacy.					
D7. When I'm down and out, I remind myself that there are lots of other people in the world feeling like I am.					
D8. When times are really difficult, I tend to be tough on myself.					

D9. When something upsets me, I try to keep my emotions in balance.					
D10. When I feel inadequate in some way, I try to remind myself that feelings of inadequacy are shared by most people.					
D11. I'm intolerant and impatient towards those aspects of my personality I don't like.					
D12. When I'm going through a very hard time, I give myself the caring and tenderness I need.					
D13. When I'm feeling down, I tend to feel like most other people are probably happier than I am.					
D14. When something painful happens, I try to take a balanced view of the situation.					
D15. I try to see my failings as part of the human condition.					
D16. When I see aspects of myself that I don't like, I get down on myself.					
D17. When I fail at something important to me, I try to keep things in perspective.					
D18. When I'm really struggling, I tend to feel like other people must be having an easier time of it.					
D19. I'm kind to myself when I'm experiencing suffering.					
D20. When something upsets me, I get carried away with my feelings.					
D21. I can be a bit cold-hearted towards myself when I'm experiencing suffering.					
D22. When I'm feeling down, I try to approach my feelings with curiosity and openness.					
D23. I'm tolerant of my own flaws and inadequacies.					
D24. When something painful happens, I tend to blow the incident out of proportion.					
D25. When I fail at something that's important to me, I tend to feel alone in my failure.					
D26. I try to be understanding and patient towards those aspects of my personality I don't like.					

Section E: Euthanasia Distress Scale (EDS)

1	2	3	4	5
Strongly disagree	Disagree	Neutral	Agree	Strongly agree

	1	2	3	4	5
E1. It is difficult to hide one's emotions when performing euthanasia on a laboratory or livestock animal.					
E2. Performing euthanasia is emotionally draining.					
E3. Thinking about performing euthanasia makes me anxious.					
E4. It would be emotionally difficult for me to euthanize an animal who has severe behavior problems but is otherwise healthy.					
E5. It would be emotionally difficult for me to euthanize an animal who has difficulty walking.					
E6. It would be emotionally difficult for me to euthanize an animal to help control the population in an animal shelter.					

E7. The physical act of performing the euthanasia is disturbing to me (e.g., administering the IV).					
E8. It would bother me if an animal I were euthanizing vocalized when I administered the IV.					

Section F: The Entrapment Short-Form Scale (E-SF)

You will now be shown some more statements about thoughts and feelings. Please read each and indicate how much it applies to you.

0	1	2	3	4
Not at all like me	A little bit like me	Moderately like me	Quite a bit like me	Extremely like me

	0	1	2	3	4
F1. I often have the feeling that I would just like to run away.					
F2. I feel powerless to change things.					
F3. I feel trapped inside myself.					
F4. I feel I'm in a deep hole I can't get out of.					

Section G: Fearlessness of death of the veterinary profession

1	2	3	4	5
Strongly disagree	Disagree	Neutral	Agree	Strongly agree

	1	2	3	4	5
G1. I value longevity of human life more than the quality of living.					
G2. My involvement in animal euthanasia acquired my concept of human's life and death.					
G3. My frequent encounter of open wounds and animal corpse had made me less anxious of death.					
G4. My involvement with animal euthanasia acquired my concept of taking one's own life.					
G5. Any form of suicide is morally wrong.					
G6. I am not at all afraid to die if I knew it won't be painful.					
G7. I worry about my family, my friends, and my pets who depends upon me.					
G8. I would recommend euthanasia for my clients when their pets were experiencing unbearable suffer.					
G9. I don't get stressed when performing euthanasia on animals.					
G10. I would not want people to think I cannot cope my life.					
G11. I would rather end my life if I happened to experience unbearable suffer.					

Section H: Patient Health Questionnaire 9 (PHQ-9)

Over the **last 2 weeks**, how often have you been bothered by any of the following problems?

0	1	2	3

Not at all	Several days	More than half the days	Nearly every day

	0	1	2	3
H1. Little interest or pleasure in doing things.				
H2. Feeling down, depressed, or hopeless.				
H3. Trouble falling or staying asleep, or sleeping too much.				
H4. Feeling tired or having little energy.				
H5. Poor appetite or overeating.				
H6. Feeling bad about yourself or that you are a failure or have let yourself or your family down.				
H7. Trouble concentrating on things, such as reading the newspaper or watching television.				
H8. Moving or speaking so slowly that other people could have noticed. Or the opposite being so figety or restless that you have been moving around a lot more than usual.				
H9. Thoughts that you (I) would be better off dead, or of hurting (my)yourself.				

Section I: Generalized Anxiety Disorder Screener (GAD-7)
Over the **last 2 weeks**, how often have you been bothered by the following problems?

1	2	3	4
Not at all	Several Days	More than half the days	Nearly every day

	0	1	2	3
I1. Feeling nervous, anxious or on edge.				
I2. Not being able to stop or control worrying.				
I3. Worrying too much about different things.				
I4. Trouble relaxing.				
I5. Being so restless that it is hard to sit still.				
I6. Becoming easily annoyed or irritated.				
I7. Feeling afraid as if something awful might happen.				

I8. If you checked off any problems, how difficult have these problems made it for you to do your work, take care of things at home, or get along with other people?

Not difficult at all	Radio button
Somewhat difficult	Radio button
Very difficult	Radio button
Extremely difficult	Radio button

Section J: Suicide Behaviors Questionnaire – Revised (SBQ-R)

J1. Have you ever thought about or attempted to kill yourself?

Never	Radio button
It was just a brief passing thought	Radio button
I have had a plan at least once to kill myself but did not try to do it	Radio button
I have had a plan at least once to kill myself and really wanted to die	Radio button

I have attempted to kill myself, but did not want to die	Radio button
I have attempted to kill myself, and really hoped to die	Radio button

J2. How often have you thought about killing yourself in the past year?

Never	Radio button
Rarely (1 time)	Radio button
Sometimes (2 times)	Radio button
Often (3-4 times)	Radio button
Very often (5 or more times)	Radio button

J3. Have you ever told someone that you were going to attempt suicide, or that you might do it?

No	Radio button
Yes, at one time, but did not really want to die	Radio button
Yes, at one time, and really wanted to do it	Radio button
Yes, more than once, but did not want to do it	Radio button
Yes, more than once, and really wanted to do it	Radio button

J4. How likely is it that you will attempt suicide in the future?

Never	Radio button
No chance at all	Radio button
Rather unlikely	Radio button
Unlikely	Radio button
Likely	Radio button
Rather likely	Radio button
Very likely	Radio button

Section K: Cyberbullying

Please select the appropriate box that best describe your cyberbullying experiences in the past 6 months.

	Yes	No
K1. I have been threatened / harassed by pet owner face-to-face, that they would post negative comments of me on social media.		
K2. I have been accused / criticised on social media by pet owner(s) for something untrue regarding veterinary medical services I provided.		
K3. I have been threatened / harassed / humiliated by pet owner(s) on social media.		
K4. I have been harassed / humiliated which involved unwanted livestreamed / video-recording made public in social media, with regard to veterinary medical services I provided.		
K5. Something negative about me had been posted by pet owner(s) in ways that I can be identified.		
K6. Someone I know in the vet profession have been criticised of on social media for veterinary medical services they might have provided.		
K7. Someone I know in the vet profession have been threatened / harassed / humiliated on social media.		

ASK only if K2 or K3 or K4 or K5 answered YES

1	2	3	4
Never	Rarely	Sometimes	Always

	1	2	3	4
K8. (if K2 or K3 or K4 or K5 code Yes) I worry about my personal safety because of negative comments made about me on social media.				
K9. (if K2 or K3 or K4 or K5 code Yes) I worry about what my current clients / other pet owners would think of me after having read negative comments of me posted on social media.				
K10. (if K2 or K3 or K4 or K5 code Yes) I worry what other veterinarians think of me after having read negative comments of me posted on social media.				

Section L: Macro-environmental changes

Consider the times of the social unrest (since 2019) and the pandemic (before vaccines were available at Feb-2021), to what extend do you agree with the statements below?

1	2	3	4	5
Strongly Disagree	Disagree	Neither agree nor disagree	Agree	Strongly Agree

During the social unrest and the pandemic, I find that…

	1	2	3	4	5
L1. Clients were more difficult to deal with.					
L2. We had more new clients and patients in the clinic.					
L3. There were more cost-related complaints.					
L4. More clients were prone to euthanise their pets.					
L5. More clients delayed their pet's regular check-ups.					
L6. My colleagues were more stressed.					
L7. We were losing harmony in our workplace.					
L8. More clients delayed their pet's essential medical check-ups					
L9. We had more cases of non-life-threatening illnesses in our clinic					
L10. More clients have spent less on treatment					
L11. More clients surrender their pets					
L12. I was more stressed, in general					
